# Correlation of plasma metabolites with glucose and lipid fluxes in human insulin resistance

**DOI:** 10.1002/osp4.402

**Published:** 2020-02-07

**Authors:** Annick V. Hartstra, Pieter F. de Groot, Diogo Mendes Bastos, Evgeni Levin, Mireille J. Serlie, Maarten R. Soeters, Ceyda T. Pekmez, Lars O. Dragsted, Mariette T. Ackermans, Albert K. Groen, Max Nieuwdorp

**Affiliations:** ^1^ Department of Internal and Vascular Medicine Amsterdam University Medical Centers Amsterdam the Netherlands; ^2^ Department of Endocrinology and Metabolism Amsterdam University Medical Centers Amsterdam the Netherlands; ^3^ Department of Nutrition, Exercise and Sports University of Copenhagen Copenhagen Denmark; ^4^ Endocrine Laboratory, Department of Clinical Chemistry Amsterdam University Medical Centers Amsterdam the Netherlands; ^5^ Department of Laboratory Medicine, University of Groningen University Medical Center Groningen the Netherlands

**Keywords:** citrulline, human insulin resistance, plasma metabolites, stable isotope hyperinsulinemic clamp

## Abstract

**Objective:**

Insulin resistance develops prior to the onset of overt type 2 diabetes, making its early detection vital. Direct accurate evaluation is currently only possible with complex examinations like the stable isotope‐based hyperinsulinemic euglycemic clamp (HIEC). Metabolomic profiling enables the detection of thousands of plasma metabolites, providing a tool to identify novel biomarkers in human obesity.

**Design:**

Liquid chromatography mass spectrometry–based untargeted plasma metabolomics was applied in 60 participants with obesity with a large range of peripheral insulin sensitivity as determined via a two‐step HIEC with stable isotopes [6,6‐^2^H_2_]glucose and [1,1,2,3,3‐^2^H_5_]glycerol. This additionally enabled measuring insulin‐regulated lipolysis, which combined with metabolomics, to the knowledge of this research group, has not been reported on before.

**Results:**

Several plasma metabolites were identified that significantly correlated with glucose and lipid fluxes, led by plasma (gamma‐glutamyl)citrulline, followed by betaine, beta‐cryptoxanthin, fructosyllysine, octanylcarnitine, sphingomyelin (d18:0/18:0, d19:0/17:0) and thyroxine. Subsequent machine learning analysis showed that a panel of these metabolites derived from a number of metabolic pathways may be used to predict insulin resistance, dominated by non‐essential amino acid citrulline and its metabolite gamma‐glutamylcitrulline.

**Conclusion:**

This approach revealed a number of plasma metabolites that correlated reasonably well with glycemic and lipolytic flux parameters, measured using gold standard techniques. These metabolites may be used to predict the rate of glucose disposal in humans with obesity to a similar extend as HOMA, thus providing potential novel biomarkers for insulin resistance.

## 1 INTRODUCTION

Obesity is often accompanied by metabolic disorders such as dyslipidemia and insulin resistance, both part of the metabolic syndrome, which in turn is a major risk factor for type 2 diabetes (T2DM), cardiovascular pathology, non‐alcoholic fatty liver disease and different types of cancer.^1^, ^2^ Insulin resistance develops prior to the beginning of overt T2DM, making its early detection of vital clinical importance. However, direct accurate evaluation of insulin resistance in relation to fasting insulin and (compensatory) hyperinsulinemia is currently only possible using complex, invasive and time‐consuming examinations such as the two‐step stable isotope based hyperinsulinemic euglycemic clamp (HIEC), a method which is regarded as the gold standard. This method also allows for distinguishing between hepatic and peripheral insulin sensitivity (Rd).

An interesting developing research field in this respect is untargeted metabolic profiling, which enables the detection of in principle thousands of plasma metabolites.^3^ These metabolites are products that reflect levels of cellular (dys)function. As they are influenced by both environmental (dietary) and biological (genetic) factors, plasma metabolites may provide insight into the balance of genotype and phenotype of T2DM. Moreover, due to the unbiased nature that characterizes metabolomic platforms, they can provide a tool to unveil novel underlying mechanisms of insulin resistance, metabolic syndrome and its dire consequences in humans with obesity.

Branched chain amino acids and other plasma metabolites such as glycerol, α‐hydroxybutyrate and mannitol^4^ are increased in patients with T2DM and might serve as potential novel biomarkers for insulin resistance.^5^, ^6^, ^7^, ^8^ However, there is a lack of studies in humans with metabolic syndrome that correlate novel metabolites with the gold standard measurement of glucose fluxes in the HIEC and, to the knowledge of this research group, no studies are available that combine both parameters. Consequently, this results in a lack of novel biomarkers able to predict aberrant glycemic control in patients with insulin resistance in an earlier phase.^9^ A recent systematic review evaluated metabolite markers identified using high‐throughput metabolomics techniques in patients cohorts with prediabetes and T2DM; however, in these patients, insulin resistance was determined via simple or indirect tests such as the oral glucose test, but never by HIEC.^8^ Alternative methods have been developed such as the Homeostatic Model Assessment (HOMA) and Quantitative insulin sensitivity check index (QUICKI), which calculate insulin resistance fairly accurately using fasting insulin and glucose concentrations. Nevertheless, as surrogate indirect methods, they have their limitations compared with the direct clamp method and are still unable to detect early stages of aberrant glucose metabolism and insulin resistance.^10^

Thus, in order to determine plasma metabolites, which are able to predict insulin resistance in an earlier stage and explore their interaction with glucose and lipid fluxes in the fasting state, liquid chromatography mass spectrometry (LC‐MS)–based untargeted plasma metabolomics (Metabolon) was applied in 60 participants with obesity with an extensive range of Rd as determined by a two‐step HIEC with stable isotopes [6,6‐^2^H_2_]glucose and [1,1,2,3,3‐^2^H_5_]glycerol, which also allowed for the measurement of insulin regulated lipolysis (Ra). Other metabolic parameters determined were hepatic insulin sensitivity, as measured via suppression of endogenous glucose production (EGP), and resting energy expenditure (REE), measured by indirect calorimetry, as well as dietary intake. This study shows that a panel of plasma metabolites could be used to predict (peripheral) insulin resistance as a substitute for the invasive laborious HIEC. Indeed, several metabolites were identified that significantly correlated with glucose and lipid fluxes, led by plasma citrulline and gamma‐glutamylcitrulline, followed by plasma betaine, beta‐cryptoxanthin, fructosyllysine, octanylcarnitine, sphingomyelin (d18:0/18:0, d19:0/17:0) and thyroxine.

## 2 METHODS

### 2.1 Study participants

Male (n=30) and female participants (n=30) with obesity, defined as body mass index (BMI) ≥ 30 kg m^−2^, were recruited via local advertisements. Participants were excluded in case of a primary lipid disorder, childhood onset obesity (due to higher risk of developing T2DM also based on more genetic predisposition), use of exogenous insulin, all medical and psychiatric conditions except for obesity related diseases, coagulation disorders, uncontrolled hypertension, renal insufficiency, substance abuse (nicotine or drugs, alcohol >2 units day^−1^), pregnancy and breastfeeding. All participants provided written informed consent, and all study procedures were approved by the Academic Medical Center IRB and conducted in accordance with the Declaration of Helsinki.

### 2.2 Study design

Following informed consent and screening, participants visited the clinical research unit after an overnight fast. Participants were instructed to record food intake and dietary habits online (mijn.voedingscentrum.nl/nl/eetmeter) the week before the study visit. During the study day, blood samples were drawn followed by the two‐step HIEC during which glucose and lipid fluxes were determined as well as REE, as described below.

## 3 MEASUREMENTS

### 3.1 Two‐step HIEC and REE

REE was measured in all participants during the basal state of the HIEC by indirect calorimetry. During 20 minutes, oxygen consumption and CO_2_ production were measured continuously using a ventilated hoodsystem (Vmax Encore 29; SensorMedics, Anaheim, CA). REE was then calculated from oxygen consumption and carbon dioxide production.^11^ To measure insulin resistance, a two‐step HIEC was performed.^12^ After an overnight, fast participants visited the hospital where they received two catheters in the peripheral veins of both arms. One catheter was used to infuse the [6,6‐^2^H_2_]glucose and [1,1,2,3,3‐^2^H_5_]glycerol (99% enriched; Cambridge Isotopes, Andover, MA, USA), together with 20% glucose enriched with 1% [6,6‐^2^H_2_]glucose and insulin (Actrapid; Novo Nordisk Farma, Alphen aan de Rijn, The Netherlands). The other catheter was used for sampling blood, which was arterialized by heating the arm with a heated‐hand box at 57°C. At 2 hours before starting the clamp (*t*=−2 h), a primed continuous infusion of both [6,6‐^2^H_2_]glucose and [1,1,2,3,3‐^2^H_5_]glycerol was started and continued until the end of the experiment. After 2 hours (*t*=0), infusion of insulin was started at a rate of 20 mU·m^−2^ (body surface area)·minute^−1^. Plasma glucose was measured every 10 minutes using a glucose analyser (YSI 2300 Stat Plus Glucose Lactate Analyzer, YSI Life Sciences, Yellow Springs, Ohio). In order to keep plasma glucose at 5 mmol·L^−1^, 20% glucose enriched with 1% [6,6‐^2^H_2_]glucose was infused at a variable rate. Insulin infusion was increased after 2 hours of insulin infusion (*t*=2 h) to 60 mU·m^−2^·minute^−1^. At *t*=0, 2 and 4 hours, five blood samples were taken to assess glucose and glycerol enrichments. Rates of disposal (Rd) of glucose and rates of appearance (Ra) of glycerol were calculated using the modified forms of the Steele equations for (non)steady state measurements as described previously.^13^

### 3.2 Biochemistry

Fasting glucose (Hitachi), insulin (Diagnostic products) and C‐reactive protein (CRP, Roche, Switzerland) were determined in fasted plasma samples. Total cholesterol, high‐density lipoprotein cholesterol (HDLc) and triglycerides (TG) were determined in EDTA‐containing plasma using commercially available enzymatic assays (Randox, Antrim, UK and DiaSys). All analyses were performed using a Selectra autoanalyser (Sopachem, The Netherlands). Low‐density lipoprotein cholesterol (LDLc) was calculated using the Friedewald formula. Insulin was determined on an Immulite 2000 system (Diagnostic Products, Los Angeles, CA, USA). As described previously, plasma analyses were performed,^14^ [6,6‐^2^H_2_] glucose enrichment was measured^15^ and [1,1,2,3,3‐^2^H_5_]glycerol determined.^16^

### 3.3 Metabolomic profiling

As mentioned above, EDTA plasma samples were taken from participants before the clamp in the fasting state. Plasma metabolite untargeted profiling analysis was carried out on plasma by Metabolon (Durham, NC), using ultrahigh‐performance liquid chromatography coupled to tandem mass spectrometry, as previously described.^17^ This method allowed for the study of 934 annotated plasma metabolites. Raw data were normalized to account for interday measurement differences. Then, each biochemical was rescaled to set the median equal to 1. Missing values, generally due to the sample measurement falling below the limit of detection, were then imputed with the minimum observed value.

### 3.4 Statistical analysis

Univariate (Spearman's rank) analyses were performed to determine which metabolites correlated significantly (*P*‐value of <.05) with Rd, EGP suppression, REE and glycerol suppression of the included participants. Subsequent multivariate linear regression was used to correct for age and BMI.

### 3.5 Machine learning to discern relative importance of plasma metabolites with Rd

In the multivariate analysis, the model was a gradient boosting regressor,^18^ which was optimized for R2, taking the previously analysed metabolic biomarkers which were correlated with Rd as the input and the Rd derived from the HIEC as the output value. To avoid overfitting, a tenfold stratified cross validation was performed over the training partition of the data (80%), while the remaining data (20%) were used as the test dataset. The identification of the most predictive plasma biomarkers was achieved through the use of permutation importance. First, a predictive model was build using the complete set of the metabolites. This led to a baseline R2 predictive performance of the model. Afterwards, the values of one metabolite were shuffled for all participants in the database and the predictive performance of the model was recomputed. The difference between the baseline performance of the model and the one computed on the shuffled dataset gave the predictive power of this particular metabolite. This procedure was then repeated for all the metabolites in the database, thus obtaining the final ranking. Moreover, a rigorous stability selection procedure was conducted to ensure the reliability and robustness of these biomarker signatures. This was repeated 20 times, and the R2 scores were computed each time and averaged for the final test R2 score. The software tools used were Python v. 3.7 (www.python.org), with the packages Numpy, Scipy and Scikit‐learn for machine learning models. To generate the visualizations, the packages used were matplotlib and plotly.

## 4 RESULTS

### 4.1 Baseline characteristics

In total, 60 participants were included with obesity (BMI ≥ 30 kg m^−2^), of which 30 male and 30 female, aged 51 ± 12 (mean ± SD) years with a BMI of 39 [34‐44] (median [interquartile range]) kg m^−2^. Metabolic baseline characteristics included a fasting glucose of 5.3 ± 0.8 mmol L^−1^, fasting insulin of 115 ± 56 pmol L^−1^ and HOMA‐IR of 3.9 ± 2.1, while total cholesterol was 5.1 ± 1.2 mmol L^−1^ with an LDL of 3.2 ± 1.1 mmol L^−1^ and HDL of 1.3 ± 0.3 mmol L^−1^. Dietary data included a caloric intake of 1,670 ± 394 kcal per day, with an intake of 66 ± 20, 79 ± 18, 16 ± 5 and 170 ± 46 g of fat, protein, fibre and carbohydrate respectively.

### 4.2 Glucose and lipid fluxes

**Figure 1:**
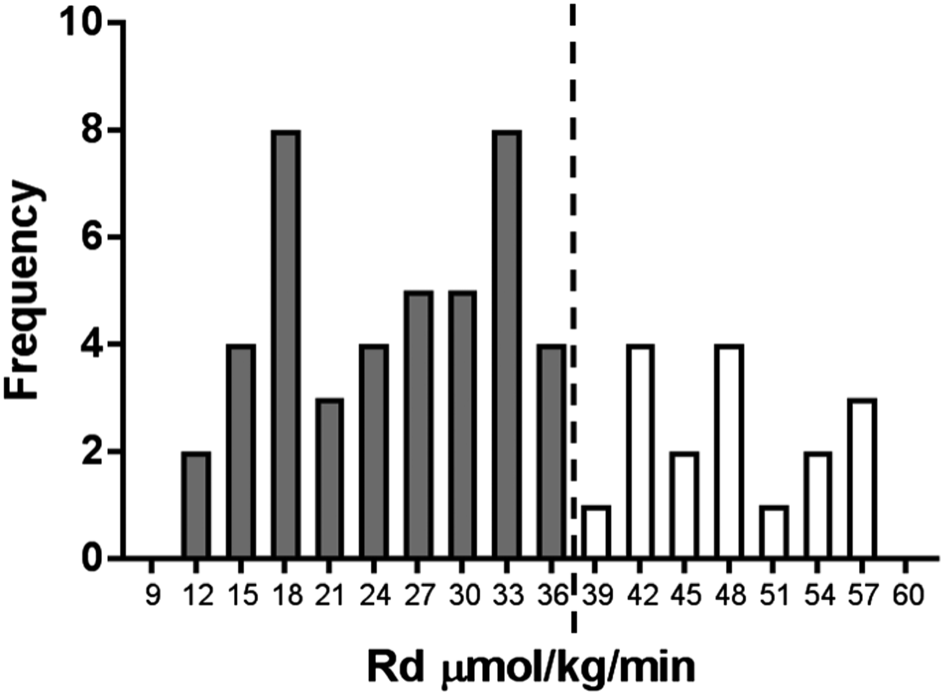
The distribution of peripheral insulin sensitivity (Rd) in all 60 participants as measured via hyperinsulinemic euglycemic clamp. The dashed line represents the cut‐off point of an Rd of 37.3 μmol kg^−1^ minute^−1^, below of which is considered insulin resistant

Insulin sensitivity was determined in each subject via a two‐step HIEC. The use of [6,6‐^2^H_2_]glucose infusion enabled the assessment of the ability of insulin to suppress EGP (EGP suppression, a marker of hepatic insulin sensitivity) and whole‐body glucose rate of disposal (Rd, a marker of peripheral insulin sensitivity). The measured Rd values ranged from high to low showing a spectrum of insulin sensitivity from insulin sensitive (maximum 57.4 μmol kg^−1^ minute^−1^) to insulin resistant (minimum 11.9 μmol kg^−1^ minute^−1^), with a mean of 31.8 ± 12.7 μmol kg^−1^ minute^−1^. EGP suppression ranged from 47 to 100 % with a mean of 75.7 ± 14 %. Other metabolic parameters measured during the clamp were REE via calorimetry, with a mean of 1,885 ± 376 kcal day^−1^, and insulin‐regulated lipolysis using [1,1,2,3,3‐^2^H_5_]glycerol to determine suppression of glycerol rate of appearance (Ra, a measure of adipose tissue insulin sensitivity) with a mean of 56.2 ± 14.3 %. As previously described, insulin resistance was defined as an Rd value of <37.3 μmol kg^−1^ minute^−1^.^19^ In the current study, population 71.7 % of all participants had an Rd lower than 37.3 and were therefore considered insulin resistant (Figure 1).

### 4.3 Correlations of metabolic parameters with plasma metabolites

**Figure 2:**
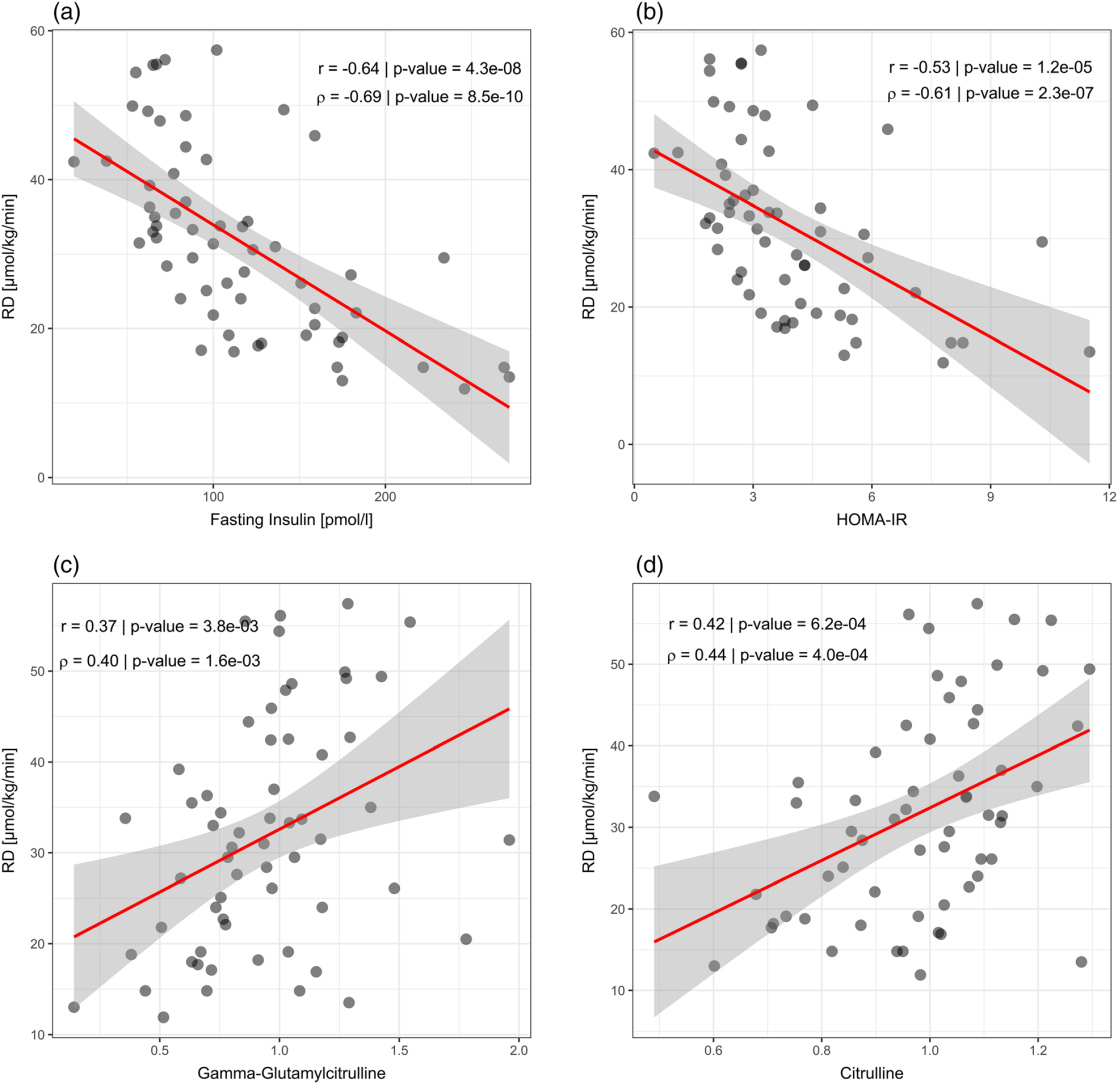
Correlation plots of most important plasma markers with peripheral insulin sensitivity (Rd). The figures show the correlation of fasting plasma insulin (A), HOMA (B), gamma‐glutamylcitrulline (C) and citrulline (D with peripheral insulin sensitivity Rd as measured by stable isotope based hyperinsulinemic euglycemic clampin 60 participants

As expected, fasting plasma insulin levels and HOMA values correlated significantly with Rd (Figures 2A and 2B). Moreover, other univariate analyses revealed significantly correlated plasma metabolites with peripheral insulin sensitivity (Rd), hepatic insulin sensitivity (EGP suppression), lipolysis (Ra), fasting insulin, HOMA and REE. Subsequent multivariate regression analyses were used to correct for the influence of age and BMI. Additionally, correlating plasma metabolites were tested for predictive ability of Rd. Seventeen plasma metabolites were found that significantly correlated with Rd, of which 12 correlated with 1 or more other fluxes and/or were found to be predictive of Rd. This included 1‐palmitoyl‐2‐stearoyl‐GPC (16:0/18:0), beta‐cryptoxanthin, betaine, fructosyllysine, glycosyl‐*N*‐(2‐hydroxynervonoyl)‐sphingosine (d18:1/24:1(2OH)), octanylcarnitine, S‐methylmethionine, sphingomyelin (d18:0/18:0, d19:0/17:0), taurodeoxycholate 3‐sulfate and thyroxine (Table 1), whereas plasma gamma‐glutamylcitrulline (Figure 2C) and citrulline (Figure 2D) were most significantly correlated with Rd. Moreover, 8 metabolites correlated significantly with EGP suppression, of which 4 correlated with Rd, 2 also with the other fluxes and 2 were predictive of Rd: beta‐cryptoxanthin, betaine, fructosyllysine and taurodeoxycholate 3‐sulfate (Table 2). Finally, 3 metabolites correlated significantly with REE of which beta‐cryptoxanthin correlated likewise with Rd and EGP suppression and was found to be predictive of Rd (Table 3). With respect to lipid fluxes, 20 metabolites correlated significantly with glycerol suppression and 6 also correlated with Rd, other fluxes or were predictive of Rd: betaine, citrulline, gamma‐glutamylcitrulline, glycosyl‐*N*‐(2‐hydroxynervonoyl)‐sphingosine (d18:1/24:1(2OH)), sphingomyelin (d18:0/18:0, d19:0/17:0) and thyroxine (Table 4).

**Table 1:** Correlations between fasting plasma metabolite and peripheral insulin sensitivity (Rd)

Metabolite	Pathway	*P*	rho	*r*	Match Other Flux	Relative Importance for Predicting Rd, %
1‐palmitoyl‐2‐linleoyl‐GPC (16:0/18:2)	Phospholipid metabolism	.03	−0.27	−0.24		
1‐palmitoyl‐2‐stearoyl‐GPC (16:0/18:0)	Phospholipid metabolism	.04	0.46	0.48		21
Beta‐cryptoxanthin	Vitamin A metabolism	.02	0.39	0.46	EGP/REE	20
Betaine	Glycine, serine and threonine metabolism	.01	0.39	0.39	EGP/Ra	19
Citrulline	Urea cycle; arginine and proline metabolism	.01	0.44	0.42	Ra	77
Cortisol	Corticosteroid	.01	−0.30	−0.31		
Decanoylcarnitine (C10)	Fatty acid metabolism (acyl carnitine, medium chain)	.01	−0.36	−0.36		
Fructosyllysine	Lysine metabolism	.01	−0.37	−0.38	EGP	
Gamma‐glutamylcitrulline	Citrulline metabolism	.03	0.40	0.37	Ra	100
Glyco‐beta‐muricholate	Primary bile acid metabolism	.03	−0.31	−0.27		
Glycodeoxycholate 3‐sulfate	Secondary bile acid metabolism	.05	−0.34	−0.34		
Glycosyl‐*N*‐(2‐hydroxynervonoyl)‐sphingosine (d18:1/24:1(2OH))	Glycolipid metabolism	.00	−0.37	−0.35	Ra	
Octanoylcarnitine (C8)	Fatty acid metabolism (acyl carnitine, medium chain)	.02	−0.40	−0.15		11
S‐methylmethionine	Methionine, cysteine, SAM and taurine metabolism	.04	0.30	0.32		12
Sphingomyelin (d18:0/18:0, d19:0/17:0)	Phospholipid metabolism	.02	−0.26	−0.25	Ra	25
Taurodeoxychol 3‐sulfate	Secondary bile acid metabolism	.03	−0.39	−0.39	EGP	
Thyroxine	Thyroid	.03	−0.40	−0.41	Ra	

*Note.* Shown are significant (*P*<.05) Spearman's correlations between metabolites and Rd after correction with multivariate linear regression for age and BMI. Metabolites were considered relevant according to match with other flux, relative importance for predicting Rd via machine learning model and/or availability of literature.

Abbreviations: EGP: endogenous glucose production (hepatic insulin sensitivity); Ra: rate of appearance (glycerol suppression); Rd: rate of disappearance (peripheral insulin sensitivity); REE: resting energy expenditure.

**Table 2:** Correlations between fasting plasma metabolite and hepatic insulin sensitivity (EGP suppression)

Metabolite	Pathway	*P*	rho	*r*	Match Other Flux	Relative Importance for Predicting Rd, %
3b‐hydroxy‐5‐cholenoic acid	Secondary bile acid metabolism	.02	−0.27	−0.19		
Beta‐cryptoxanthin	Vitamin A metabolism	.02	0.35	0.28	Rd/REE	20
Betaine	Glycine, serine and threonine metabolism	.01	0.28	0.25	Rd/Ra	19
Docosatrienoate (22:3n3)	Fatty acid metabolism	.05	−0.36	−0.35		
Etiocholanolone glucuronide	Androgenic steroids	.04	−0.31	−0.33		
Fructosyllysine	Lysine metabolism	.01	−0.37	−0.36	Rd	
Lysine	Lysine metabolism	.04	−0.29	−0.32		
Taurodeoxychol 3‐sulfate	Secondary bile acid metabolism	.03	−0.27	−0.10	Rd	

*Note.* Shown are significant (*P*<.05) Spearman's correlations between metabolites and EGP suppression after correction with multivariate linear regression for age and BMI. Metabolites were considered relevant according to match with other flux, relative importance for predicting Rd via machine learning model and/or availability of literature.

Abbreviations: BMI: body mass index; EGP: endogenous glucose production.

**Table 3:** Correlations between fasting plasma metabolite and resting energy expenditure (REE)

Metabolite	Pathway	rho	*P*	*r*	Match Other Flux	Relative Importance for Predicting Rd, %
1‐docosahexaenoylglycerol (22:6)	Fatty acid metabolism	−0.43	.01	−0.44		
Beta‐cryptoxanthin	Vitamin A metabolism	−0.43	.02	−0.31	Rd/EGP	20
Docosahexaenoate (DHA; 22:6n3)	Fatty acid metabolism	−0.44	.03	−0.46		

*Note.* Shown are significant (*P*<.05) Spearman's correlations between metabolites and REE after correction with multivariate linear regression for age and BMI. Metabolites were considered relevant according to match with other flux, relative importance for predicting Rd via machine learning model and/or availability of literature.

Abbreviation: BMI: body mass index.

**Table 4:** Correlations between fasting plasma metabolite and glycerol suppression (Ra)

Metabolite	Pathway	*P*	rho	*r*	Match Other Flux	Relative Importance for Predicting Rd, %
1‐linoleoyl‐GPI* (18:2)*	Phospholipid metabolism	.00	−0.45	−0.60		
1‐oleoyl‐GPI (18:1)	Phospholipid metabolism	.03	−0,32	−0.33		
1‐palmitoleoyl‐2‐linolenoyl‐GPC (16:1/18:3)*	Phospholipid metabolism	.01	−0,31	‐0.38		
1‐palmitoyl‐2‐linoleoyl‐GPI (16:0/18:2)	Phospholipid metabolism	.04	−0,34	−0.33		
1‐stearoyl‐2‐linoleoyl‐GPI (18:0/18:2)	Phospholipid metabolism	.01	−0,30	−0.37		
3‐hydroxyoleate	Fatty acid metabolism	.03	−0,26	−0.21		
5alpha‐androstan‐3alpha,17beta‐diol monosulfate (2)	Androgenic steroids	.02	0.40	0.31		
Betaine	Glycine, serine and threonine metabolism	.01	0.40	0.39	Rd/EGP	19
Biliverdin	Hemoglobin and porphyrin metabolism	.03	0.29	0.33		
Citrulline	Urea cycle; arginine and proline metabolism	.01	0.39	0.42	Rd	77
Epiandrosterone sulfate	Androgenic steroids	.04	0.28	0.05		
Gamma‐glutamylcitrulline	Citrulline metabolism	.01	0.40	0.41	Rd	100
Glycosyl‐*N*‐(2‐hydroxynervonoyl)‐Sphingosine (d18:1/24:1(2OH))	Glycolipid metabolism	.04	−0.33	−0.28	Rd	
Guanidinoacetate	Creatine metabolism	.04	0.35	0.33		
*N*‐stearoyl‐sphinganine (d18:0/18:0)	Ceramide metabolism	.03	−0,36	−0.28		
Sphingomyelin (d18:0/18:0, d19:0/17:0)	Phospholipid metabolism	.01	−0,36	−0.33	Rd	25
Sphingomyelin (d18:1/18:1, d18:2/18:0)	Phospholipid metabolism	.02	−0,35	−0.32		
Sphingomyelin (d18:1/20:2, d18:2/20:1, d16:1/22:2)	Phospholipid metabolism	.03	−0,28	−0.27		
Sphingomyelin (d18:2/16:0, d18:1/16:1)	Phospholipid metabolism	.04	−0,31	−0.37		
Thyroxine	Thyroid	.02	−0.34	−0.40	Rd	

*Note.* Shown are significant (*P*<.05) Spearman's correlations between metabolites and Ra after correction with multivariate linear regression for age and BMI. Metabolites were considered relevant according to match with other flux, relative importance for predicting Rd via machine learning model and/or availability of literature.

Abbreviation: BMI: body mass index.

### 4.4 Combining plasma metabolites to predict peripheral insulin sensitivity (Rd)

**Figure 3:**
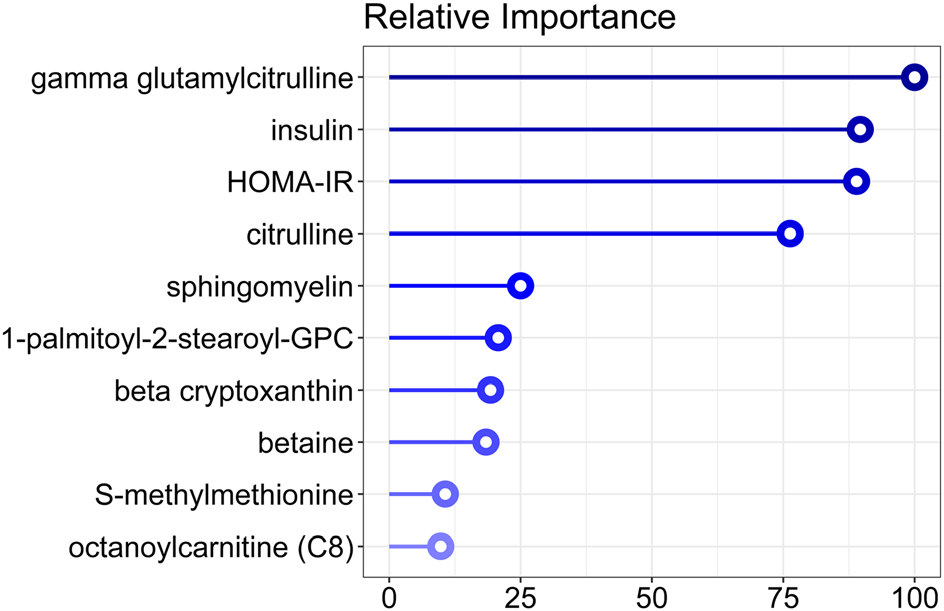
Feature importance plot of metabolites in predictive model of Rd. The figures show the top 10 metabolites that were found to be most important in the Rd predictive model. The values were weighted with the feature importance as mentioned in Section 2

The 17 metabolites found to correlate significantly with Rd originate from a number of different metabolic pathways. To investigate whether a reliable prediction of Rd could be made from a combination of these metabolites, a Gradient Boosting Machine Learning model was used.^18^ The average *R* ^2^ achieved by the model across the 20 performed shuffles was 0.24. Figure 3 shows the top 10 metabolites that were found to be most important in the Rd predictive model, headed by plasma (gamma‐glutamyl)citrulline levels, followed by HOMA and fasting insulin levels.

## 5 DISCUSSION

In this exploratory cross‐sectional study (untargeted), metabolic profiling was applied using fasting plasma samples of 60 participants with obesity with an extensive range of peripheral insulin sensitivity, revealing several metabolites that significantly correlated with glucose and lipid fluxes as determined by stable isotope‐based two‐step HIEC. Subsequent machine learning analysis showed that a combination of these plasma metabolites may potentially be used to predict peripheral insulin resistance and sensitivity (Rd) with a reasonable accuracy. Machine learning analysis on EGP and REE was not possible due to the low number of correlating parameters. However, plasma gamma‐glutamylcitrulline emerged as the most important component in the machine learning model for Rd, followed by its precursor citrulline. However, further (in vitro and in vivo) data are needed to strengthen the biological evidence of the (gamma‐glutamyl)citrulline pathway in the pathophysiology of human insulin resistance and to prove the value of this marker in its early detection.

Interestingly, citrulline and its metabolite gamma‐glutamylcitrulline have been already linked to insulin sensitivity of adipose tissue (as determined by [1,1,2,3,3‐^2^H_5_]glycerol). L‐citrulline is a non‐essential amino acid^20^ with strong antioxidant properties and in humans is involved in the urea cycle in the liver. The amino acid is not secreted by the liver, but the amount found in plasma has been shown to originate from production by enterocytes.^21^ Via its product arginine, citrulline is important in the metabolism of nitric oxide (NO), a signalling molecule critical for the cardiovascular system as a vasodilatator.^22^ Oral supplementation of citrulline is more potent at increasing serum arginine, and thus NO levels, than arginine itself due to a first‐pass extraction effect by arginase in the gastro‐intestinal tract, which does not affect citrulline.^21^ This has made it a promising focus for research into new treatment modalities for metabolic and cardiovascular‐related diseases, as endothelial dysfunction, associated with obesity‐induced insulin resistance, hypertension and impaired skeletal muscle metabolism have been linked to arginine and NO deficiency in humans.^23^, ^24^, ^25^ Supporting the observation in this study of the positive correlation between citrulline and glycerol suppression, animal studies have shown promising results of citrulline supplementation on lipolysis, reducing adipose tissue.^26^

The medium‐chain acylcarnitines, decanoylcarnitine and octanoylcarnitine were both negatively associated with insulin sensitivity, and especially octanoylcarnitine was found to be predictive of Rd (Figure 3). There is growing evidence of plasma acylcarnitines as potential biomarkers for insulin resistance.^27^ In study participants with T2DM, octanoylcarnitine and decanoylcarnitine were both increased compared with controls.^28^ A substrate overload of fatty acids may result in incomplete long‐chain fatty acid β‐oxidation, allowing for accumulation of acylcarnitines which via pro‐inflammatory NFkB‐associated pathways impair insulin action, thus promoting insulin resistance.^27^ In participants with obesity and T2DM, in whom the degree of insulin resistance was measured via a HIEC similar to this study, plasma acylcarnitines were found to be significantly elevated,^29^ supporting the current observations.

Previous data were confirmed by our results, showing an inverse significant association of plasma thyroxine levels with both peripheral insulin sensitivity and adipose tissue insulin sensitivity.^30^ Both Rd and Ra correlated negatively with sphingomyelin (d18:0/18:0,d19:0/17:0), which is in accordance with previous data in patients with TDM2.^31^ In line, a positive significant correlation was observed in this study between plasma betaine and peripheral, hepatic and adipose tissue insulin sensitivity. Betaine, also known as trimethylglycine, is an amino acid, which is derived in the human body via dietary intake or via mitochondrial oxidation of choline in kidney and liver (driving TMAO production),^32^, ^33^ and low betaine is significantly associated with key criteria of metabolic syndrome.^34^, ^35^ Moreover, in the current study, the significant positive correlation was confirmed between plasma beta‐cryptoxanthin (β‐CRX, a carotenoid) and peripheral and hepatic insulin sensitivity.^36^, ^37^ In obese animal models, β‐CRX supplementation was inversely related to the risk of insulin resistance and liver dysfunction, possibly mediated by inhibiting inflammatory gene expression via the suppression of macrophages and of the signalling of TNFα and gut‐derived endotoxins (LPS).^38^, ^39^, ^40^ In humans, serum β‐CRX was likewise inversely related to insulin resistance^41^ and decreased amount of visceral fat.^42^ Finally, the negative correlation between fructosyllysine and both Rd and REE is in agreement with other studies where the increase of this metabolite has been associated with obesity and insulin resistance in humans.^43^ Fructosyllysine is a glyco‐amino acid, also known as an early glycation product, which is directly toxic to tissue and is a precursor of advanced glycation end product formation.^44^, ^45^

In conclusion, unravelling the interaction between plasma metabolites with glucose and lipid fluxes in persons with insulin resistance is essential for our understanding of its pathophysiology and uncovering yet unknown biomarkers and novel treatments. By combining targeted plasma metabolomics with gold standard stable isotope‐based glucose and lipid fluxes in participants with obesity with an extensive range of Rd, some well‐known and some less known indicators are provided for insulin resistance and a panel of these metabolites is shown that may possibly be used to predict Rd to a similar extend as fasting insulin and HOMA. Thus, plasma levels of (gamma‐glutamyl)citrulline may serve as a viable candidate for early clinical diagnosis of insulin resistance (eg, in combination with HOMA) when a stable isotope‐based HIEC is not available.

There were some limitations to this study. First, only a markedly obese population was studied and applicability of results to patients who are less obese remains open. Also, based on the nature of targeted metabolomics, plasma (gamma‐glutamyl)citrulline levels were measured in a semi‐quantitative fashion. Finally, the sample size of this pilot study was relatively small, but to the knowledge of this research group, this is the only study that compares gold standard stable isotope‐based HIEC to plasma metabolites. Moreover, both multivariate as well as machine learning approach analyses were performed for non‐linear multi‐variate analysis of the metabolites in various combinations (19), thus aiming to control for type 1 errors as much as possible. Nevertheless, when further optimized, a biomarker panel based on citrulline derivatives (presumably in combination with HOMA) may replace the expensive and laborious HIEC to estimate Rd. Future research aimed at validating these citrulline biomarkers in larger prospective cohorts will have to confirm the findings reported here, yet the identified plasma metabolites could have the potential to enhance early identification of individual risks in patients at risk for T2DM and monitoring of treatment response.

## AUTHOR TASKS

AVH, PFdG, AKG and MN were responsible for the study design; AVH, MJS, MRS, CTP, LOD and MTA conducted data collection; AVH, EL, PFdG and DMB conducted data analysis; and AVH, AKG and MN drafted the manuscript.

## CONFLICTS OF INTEREST

None to declare.
